# The Influence of CaO and MgO on the Mechanical Properties of Alkali-Activated Blast Furnace Slag Powder

**DOI:** 10.3390/ma15176128

**Published:** 2022-09-03

**Authors:** Shihui Feng, Jing Zhu, Ruixuan Wang, Zijian Qu, Lizhuo Song, Hui Wang

**Affiliations:** 1 College of Civil Engineering and Architecture, Harbin University of Science and Technology, Harbin 150080, China; 2 School of Civil and Environmental Engineering, Ningbo University, Ningbo 315000, China

**Keywords:** flexural strength, compressive strength, alkali-activated blast furnace slag powder, dry shrinkage rate, MgO, CaO

## Abstract

CaO and MgO are both reported as effective activators for blast furnace slag. However, the synergistic effect of these two components on the mechanical properties of alkali-activated blast furnace slag remains unclear. In this study, the flexural and compressive strengths of alkali-activated blast furnace slag powder with MgO and CaO range from 0% to 30% by the mass ratio of alkali-activated blast furnace slag powder are investigated. Moreover, the dry shrinkage rate of alkali-activated blast furnace slag powder is measured. One percent refractory fibers by volume of binder materials are added in the alkali-activated blast furnace slag. Some refractory fibers are treated with water flushing, meanwhile, some refractory fibers are directly used without any treatment. Finally, the scanning electron microscope, the thermogravimetric analysis curves and the XRD diffraction spectrums are obtained to reflect the inner mechanism of the alkali-activated blast furnace slag powder’s mechanical properties. The water-binder ratios of the alkali-activated blast furnace slag powder are 0.35 and 0.42. The curing ages are 3 d, 7 d and 28 d. The measuring temperature for the specimens ranges from 20 °C to 800 °C. Results show that the flexural and compressive strengths increase with the increased curing age, the decreased water-binder ratio and the addition of refractory fibers. The water-treated refractory fibers can improve the mechanical strengths. The mechanical strengths increase in the form of a quadratic function with the mass ratio of MgO and CaO, when the curing age is 3 d, the increasing effect is the most obvious. A higher water-binder ratio leads to an increasing the drying shrinkage rate. The activated blast furnace slag powder with CaO shows a higher drying shrinkage rate. The mechanical strengths decrease with the increasing testing temperature.

## 1. Introduction

The expansion of MgO through hydration reaction has a certain compensation effect on the shrinkage of concrete. Meanwhile, the shrinkage reduction effect is more economical and effective than that of traditional additives. Therefore, MgO is commonly used in practical civil engineering [1,2]. Due to the large volume of hydration products (Mg(OH)_2_), the shrinkage of the materials can be reduced [3,4,5]. Moreover, the MgO can improve the dense of the materials. Additionally, the magnesium oxide can be used to improve the long-term volume stability of slag cement in slag system [6,7,8].

The active MgO was firstly used in ordinary Portland cement in 2001 by Harrison [9]. As reported in some journals, the MgO has been proved to be an effective slag activator [10,11,12]. MgO with high specific surface area and high reactivity can improve the compactness and strength of the cement by reducing porosity. Yi et al. [13]. found that the addition of MgO is effective to activate the mechanical property of blast furnace slag powder. In their study, the compressive strength of blast furnace slag powder with MgO cured in the standard curing environment for 28 days is 1.3 times of that with Ca(OH)_2_. Moreover, as pointed out in Jin’s study that the blast furnace slag powder with MgO shows lower 1 day’s compressive strength and higher 28 days’ compressive strength [14].

Alkali-activated slag cementitious material (AASCM) has attracted many scholars at home and abroad for its excellent mechanical properties and durability [15,16,17]. In recent years, more and more studies have been carried out to improve the properties of alkali slag cementitious materials by using magnesium oxide and fibers. It is found that rigid fibers, polypropylene fibers, carbon fibers and glass fibers are conducive to reducing shrinkage [18,19,20,21]. However, the traditional reinforced fibers are expensive, which makes high cost of the cement concrete. The refractory fibers are solid wastes without reasonable treatment [22,23]. This kind of fiber is mainly composed of alumina and zirconia, which shows good high temperature resistance. Based on these properties, the refractory fibers, the refractory fibers can be used for improving the mechanical strength and fire resistance. Aydin et al. [24] reported that alkali slag cementitious materials show excellent high temperature resistance due to lower hydrated calcium silicate gel than cement. Çelikten et al. [25] found that alkali-activated blast furnace slag powder mortar has higher high temperature resistance than Portland cement mortar at 800 °C. Rashad et al. [26] studied the influence of high temperature on the performance of alkali slag cement and found that the residual strength of alkali slag cement is higher than that of Portland cement in high temperature environment. Alkali-activated blast furnace slag powder with finer slag particle size shows better high temperature resistance.

As reported in prior research, the activation of single unit of MgO on the blast furnace slag powder is lower than the assembly unit of MgO and potassium water glass [27,28]. In order to solve the problem of large shrinkage of slag system, Puertas et al. [29] studied the effect of polypropylene fibers on the properties of alkali activated composites and found that adding 1% polypropylene fiber can reduce the shrinkage. Kai et al. [30] used MgO, CaO and MgO-CaO mixtures to excite slag, and the results showed that MgO and CaO mixtures can effectively inhibit the shrinkage of harden specimens, but the strength is lower than that of composite with potassium silicate and NaOH [31]. Zheng et al. [32] used MgO and sodium silicate to excite the slag, and the results showed that the composite excitation could form richer hydration products possessing higher mechanical strength. However, little attention has been paid to the activated effect of the assembly unit of MgO and potassium water glass on the blast furnace slag powder. According to above descriptions, CaO and MgO can both act as effective activators for blast furnace slag. However, the synergistic effect of these two components on the mechanical properties of alkali-activated blast furnace slag remains unclear. Moreover, little research about the influence of high temperature on its mechanical performance is reported.

In this paper, the mechanical strength and the drying shrinkage rate are investigated. The curing age, the water-binder ratio and the additions of MgO, CaO and refractory fibers are considered. The thermogravimetric analysis curves, scanning electron microscope (SEM) and X-ray diffraction (XRD) spectra are investigated to reflect the inner mechanism of the mechanical strengths and the dry shrinkage rate. This study will provide a new kind of cementitious material.

## 2. Experimental

### 2.1. Raw Materials

The alkali-activated blast furnace slag powder in this study is composed of the blast furnace slag powder, light-burned magnesia powder (MgO), potassium sodium silicate (K_2_SiO_3_), sodium hydroxide (NaOH), water, expansive agent and refractory fiber.

The blast furnace slag (BFS) powder is provided by Tangshan Tielan Company, Tangshan, China. The main activity indexes, the alkaline coefficient and activity coefficient of blast furnace slag powder are 1.69, 0.97 and 0.42.

The light burned magnesia powder is made of magnesite calcined at 950 °C and supplied by Liaoning Dashiqiao Tianyi Refractory Co., Ltd., Dashiqiao, China. The active content of light burned magnesia powder is 52.3%.

Potash water glass (K_2_O·n SiO_2_·H_2_O) is provided by Xingtai Dayang Chemical Co., Ltd., Xingtai, China. The initial modulus, baume degree and density of potash water glass are 2.78, 46.3 and 1.434 g/cm^3^. The chemical compositions SiO_2_ and K_2_O of potash water glass are 27.49% and 15.50%. The sodium hydroxide is provided by Tianjin Tianli Chemical Reagent Co., Ltd., Tianjin, China, with a mass ratio of ≥96.0%. The expansive agent is produced by Henan Pearl Chemical Co., Ltd. (Puyang, China) with a burning loss of 2.02. Refractory fiber is produced by Shandong Luyang Energy-saving Materials Co., Ltd. (Zibo, China), which is polycrystalline refractory fiber, including mullite fiber, alumina fiber and zirconia fiber. Table 1 shows the main chemical composition of raw materials. Additionally, the mixing proportions of alkali-activated blast furnace slag powder is shown in Table 2.

### 2.2. Specimens’ Preparation

Firstly, the potash water glass and sodium hydroxide are mixed in the NJ-160A cement paste mixer (Wuxi Jianyi instrument factory, Wuxi, China) and stirred with the speed of 140 r/min for 1 min. After this mixing is finished, the blast furnace slag powder is added to the cement paste mixer and 1 min of stirring with the speed of 140 r/min is provided. Then, the water and refractory fiber are poured into the mixer and stirred at the speed of 140 r/min for 2 min, and then, another 2 min stirring with the speed of 285 r/min is provided for the mixture. After the stirring, all fresh mixture is poured to form specimens with size 40 × 40 × 160 mm^3^. When the pouring is finished, the specimens are used for vibration and compaction by a vibrating, then the specimens are cured in the environment of 65% relative humidity and 20 °C for 1 day and then demolded. Finally, all specimens are moved to the standard curing room with relative humidity and temperature of 98.1% and 20 °C, respectively, until the required curing age.

### 2.3. Measurement Methods

#### Measurement of Mechanical Strength and Dry Shrinkage Rate

**Figure 1:**
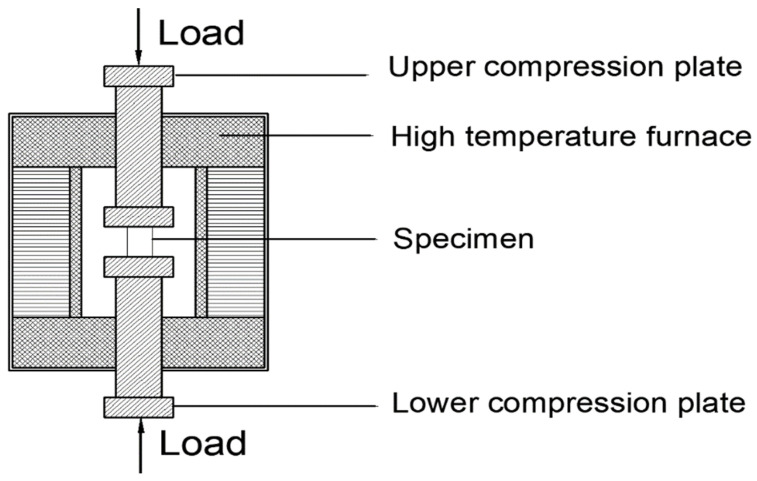
Automatic universal testing machine equipped with a temperature controllable box.

HYE-300B cement flexural and compressive constant stress testing machine produced by Beijing Sanyu Weiye, Beijing, China, is used for the measurement of mechanical strengths. The loading speeds for the compressive and flexural strengths are 2.4 kN/s and 0.05 kN/s respectively. Meanwhile, the specimens with this size are applied in the measurement of drying shrinkage. The testing methods can be found in ASTM C109/C109M-2011a ASTM International standard [33]. Automatic universal testing machine equipped with a temperature controllable box shown in Figure 1 is provided to determine the mechanical strength with different temperatures. The testing temperatures of specimens with water-binder ratio of 0.35, 10% MgO and 20% CaO by mass ratios of the total binder materials are 20 °C, 200 °C, 400 °C, 600 °C and 800 °C. Specimens are kept in these temperatures for 4 h before testing. The experimental details can be found in Feng’s research [34].

The dry shrinkage rate is carried out by the following steps.

The BC-160 type length comparator produced by Cangzhou Bowei, Cangzhou City, China is placed on a flat desktop. The top of the dial indicator is aligned with “0”. Then, the specimens are put into the length gauge and observe the dial indicator to read the length. The testing methods can be found in JGJT70-2009 Chinese standard [35].

**Figure 2:**
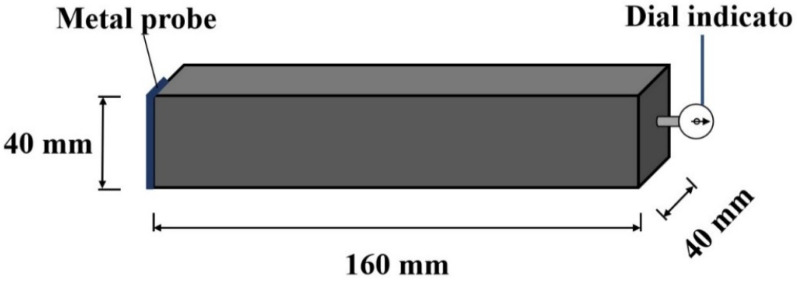
The measurement of dry shrinkage rate.

The dry shrinkage rate is calculated by Equation (1).

(1) $$\varepsilon =\frac{L_{1}-L_{t}}{L_{t}}$$

where *ε* is the dry shrinkage rate. *L*_1_ and *L_t_* are the lengths of specimen cured for 1 day and 28 days, respectively. The diagrammatic sketch of the dry shrinkage rate is illustrated in Figure 2.

### 2.4. Steps of Micro-Performance

Central crushing part of specimen is selected for the measurements of scanning electron microscope and the thermogravimetric analysis. Samples with mung bean size are vacuum sprayed with gold. After that, the samples are used for obtaining the scanning electron microscope photos by the thermal field emission scanning electron microscope SU5000 produced by Hitachi Hi Tech Co., Ltd., Toko, Japan. Meanwhile, the remaining samples are ground into a powder by a ball mill and used for obtaining the thermogravimetric curves. TGADSC3+ thermogravimetric and synchronous thermal analyzer produced by mettletoledo, Zurich, Switzerland is used for obtaining the thermogravimetric curves. The temperature for thermogravimetric test ranges from 20 °C to 1000 °C. Some powdery sample is used for the measurement of X-rays diffraction spectrum with Brooke X-ray diffractometer D2PHASER, provided by Germany Brooke company, Berlin, Germany. The measuring details can be found in Wang’s research [36].

## 3. Results and Discussion

### 3.1. Influence of Curing Age

**Figure 3:**
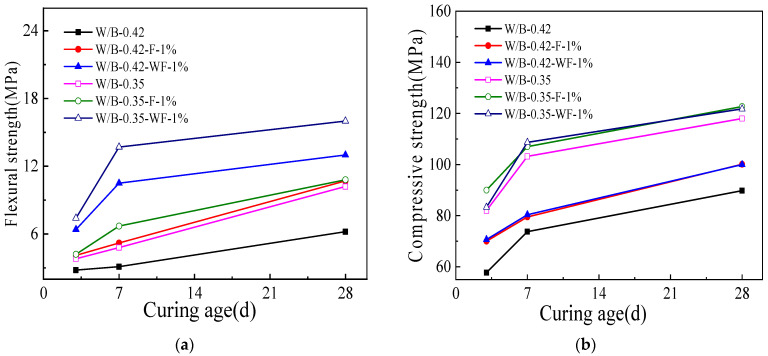
The mechanical strengths with different curing age. (**a**) Flexural strength. (**b**) Compressive strength.

Figure 3 is the flexural and compressive strengths of blast furnace slag activated by water glass and refractory fibers. It can be seen from Figure 3; the flexural and compressive strengths gradually increase with the increasing curing age. The mechanical strengths increase rapidly when the curing age ranges from 1 d to 7 d. Meanwhile, when the curing age increases from 7 d to 28 d, the flexural and compressive strengths increase slowly. This is attributed to the fact that the reactivity of the alkali activated blast furnace slag is high at early curing age and then is low at later curing age [37]. Therefore, the mechanical strengths increase rapidly at early curing age and increase slowly at later curing age [37]. Moreover, as illustrated in Figure 3, the mechanical strengths are decreased by higher water-binder ratio and increased by the addition of refractory fibers. The refractory fibers treated by water can effectively improve the flexural strength of alkali activated slag. However, the water treated refractory fibers demonstrate the negative effect on the alkali activated blast furnace slag powder. Compared with the blast furnace slag activated by only CaO or MgO, the flexural strength of this activated blast furnace slag is increased by 11.7~32.8%, and the compressive strength is increased by 15.6~37.2% [38].

### 3.2. Influence of MgO and CaO on Mechanical Strengths

**Figure 4:**
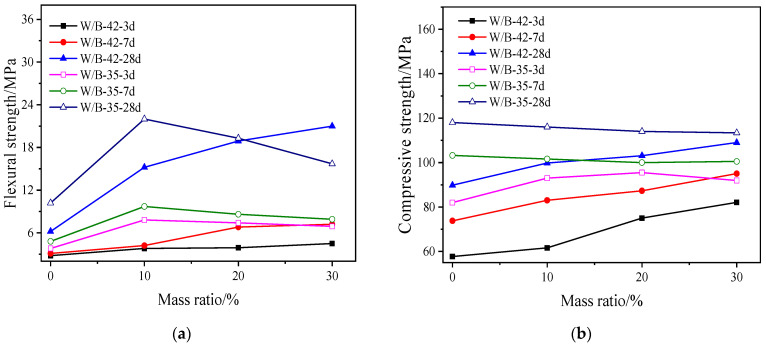
The mechanical strengths of activated blast furnace slag powder with MgO. (**a**) Flexural strength. (**b**) Compressive strength.

Figure 4 shows mechanical strengths of activated blast furnace slag powder with different dosages of MgO. As illustrated in Figure 4, the mechanical strengths of activated blast furnace slag powder increase with the increasing dosage of MgO. The enhancement effect on mechanical strengths of activated blast furnace slag powder is more obvious when the curing ages are 1 d and 3 d. This is attributed to the hydration products (hydrotalcite and hydrated magnesium silicate gel) formed by MgO and water. However, when the curing age is higher than 3 d, the mechanical strengths increase slowly. This is ascribed to the fact that the ion concentration will decrease with the increase of hydration process and the increase of gel. The increased gel may absorb on the surface of the hydration thus delaying the increase of mechanical strength [39].

**Figure 5:**
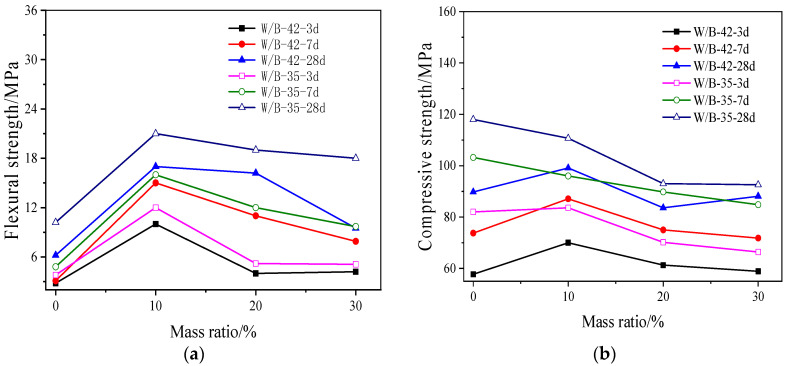
The mechanical strengths of activated blast furnace slag powder with CaO. (**a**) Flexural strength. (**b**) Compressive strength.

The compressive and flexural strengths of activated blast furnace slag powder with swelling agent which is mainly composed by CaO is shown in Figure 5. It is observed in Figure 5, the compressive and flexural strengths firstly increase with the dosage of CaO ranging from 0% to 10%. However, when the addition of CaO is higher than 10%, the compressive and flexural strengths decrease with the increasing dosage of CaO. Therefore, 10% by mass of the total binder materials is the threshold value of the strength. Moreover, the increasing curing age and lower water-binder ratio led to higher mechanical strength. The hydrolysis rate of CaO is faster than that of MgO, the addition of CaO will be rapidly hydrolyzed to form Ca(OH)_2_, which will continuously ionize Ca^2+^ and OH^−^ into the solution. High concentration of OH^−^ is activated to the dissolution of blast furnace slag powder. Small CaO content results in lower Ca/Si molar ratio inner solution. Therefore, the pH value in the early stage is increased by the addition of CaO. Consequently, the large amount of free SiO_4_^4−^ reacts with Mg^2+^ and the amount of hydrated calcium silicate increases, thus the mechanical strengths are improved. However, when the CaO content is higher than 10%, the Ca/Si molar ratio increases, and SiO_4_^4−^ preferentially forms hydrated calcium silicate with Ca^2+^, thus inhibiting the reaction between SiO_4_^4−^ and Mg^2+^, hindering the formation of hydrotalcite and hydrated magnesium silicate [40].

**Figure 6:**
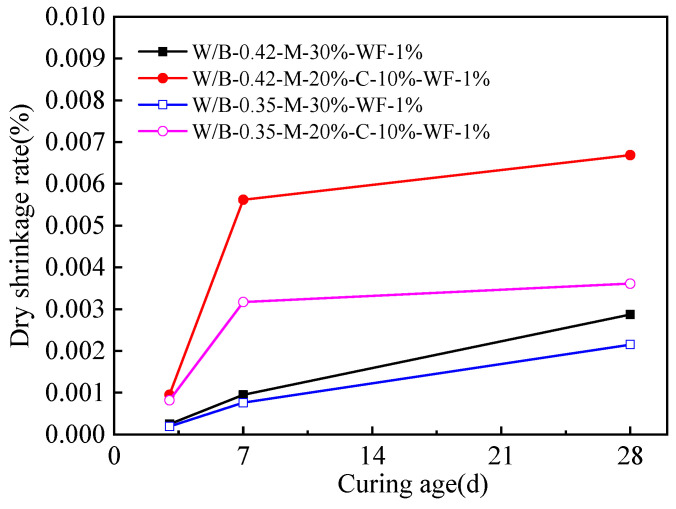
The shrinkage rate of reactive powder concrete.

Figure 6 shows the drying shrinkage rate of activated blast furnace slag powder. As shown in Figure 6, higher water-binder ratio leads to increasing the drying shrinkage rate of the activated blast furnace slag powder. This is attributed to the fact that the free water inner activated blast furnace slag powder decreases obviously due to the improvement of hydration degree by the increased curing age and higher water-binder [41]. The decreased free water leads to increasing the drying shrinkage rate. Moreover, activated blast furnace slag powder with CaO shows higher drying shrinkage rate. This is ascribed to the fact that the expanding effect of CaO is lower than that of MgO; therefore, the drying shrinkage rate of blast furnace slag powder with CaO is lower.

### 3.3. Microscopic Analysis

**Figure 7:**
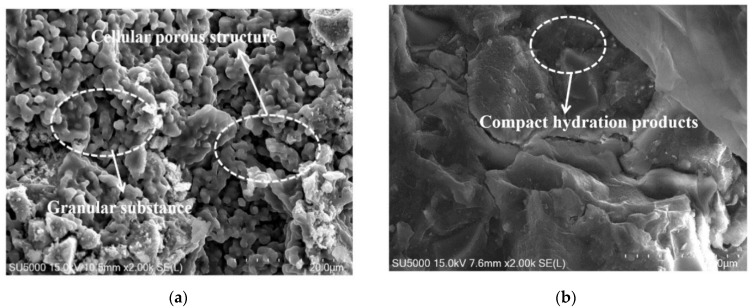

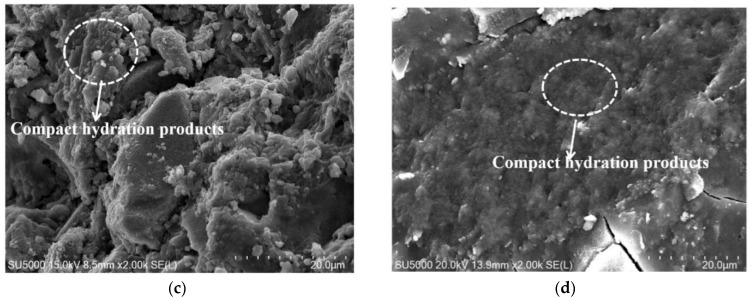
The SEM morphology of alkali-activated blast furnace slag powder with MgO. (**a**) With 10% MgO-3 d. (**b**) With 10% MgO-28 d. (**c**) With 30% MgO-3 d. (**d**) With 30% MgO-28 d.

Figure 7 shows the scanning electron microscope photos of alkali-activated blast furnace slag powder with 10% MgO and 30% MgO, respectively. The compactness of the alkali-activated blast furnace slag powder is improved when more dosage of MgO is added. This is attributed to the fact that the hydrated magnesium silicate gel formed by MgO and water can cover on the surface of the hydrated calcium silicate gel and fill the pore structure of the hydration products [42,43]. Moreover, as discovered in Figure 7, the hydration products are more compact when the curing age is higher. This is due to the fact that the with the increase of hydration degree, the pores in the cement stone will be filled gradually, and the compactness will be improved.

**Figure 8:**
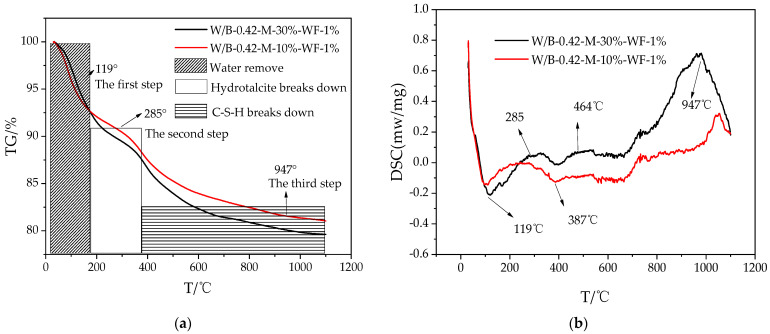
Thermogravimetric analysis curves of specimens. (**a**) TG curves. (**b**) DSC curves.

**Figure 9:**
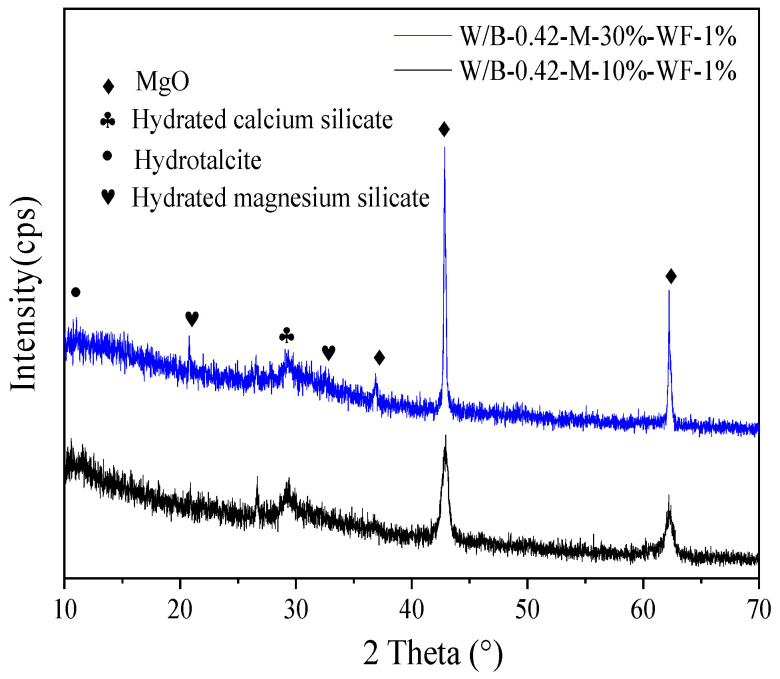
The X-ray diffraction (XRD) spectra curves.

Figure 8 shows the results of thermogravimetric analysis of alkali-activated blast furnace slag powder with 10% MgO and 30% MgO cured for 28 d. It can be observed from Figure 8, the thermogravimetry of alkali-activated blast furnace slag powder decreases with the increasing temperature. The DSC curves are shown in Figure 9. As can be observed in Figure 9, the corresponding temperatures of the endothermic peak of all the DSC curves are 119 °C, 285 °C and 947 °C. This is attributed to the fact that when the temperature ranged from 0 °C to 119 °C, the thermogravimetry decreases due to the evaporation of free water in the pore solution. Meanwhile, when the temperature increases from 119 °C to 285 °C, the thermogravimetry decreases [44]. This is ascribed to the decomposition of hydrotalcite. Moreover, when the temperature increases from 285 °C to 947 °C, the thermogravimetry decreased, this is because of the decomposition of hydrated calcium silicate [45].

The X-ray diffraction (XRD) spectra curves of specimens with 10% MgO and 30% MgO cured for 28 d are shown in Figure 9. It can be observed from the XRD spectra curves, the hydrated calcium silicate, unhydrated magnesium oxide, hydrotalcite and hydrated magnesium silicate exist in Figure 9. As illustrated in Figure 9, the hydrated magnesium silicate’s intensity of specimens with 30% MgO is higher than that of specimens with 10% MgO, which further confirms that MgO has a higher ability to promote the mechanical strengths of alkali activated blast furnace slag powder.

### 3.4. Influence of High Temperature on the Mechanical Strength

**Figure 10:**
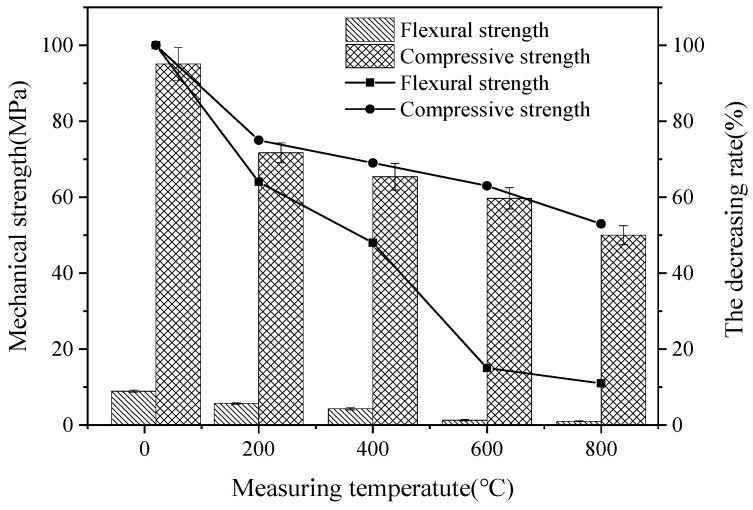
The mechanical strength with different testing temperature.

Figure 10 shows the mechanical strength of alkali-activated blast furnace slag powder with water-binder ratio of 0.35, 10% MgO and 20% CaO by mass ratios of the total binder materials. It can be observed from Figure 10, the flexural and compressive strengths decrease with the increasing temperature. This is attributed to the fact that the free water in the specimen evaporates, resulting in holes and cracks. Therefore, the stress concentration forms during the loading process, so the strength decreases significantly. Moreover, higher temperature leads to the hydrated calcium silicate gel inside the specimen decomposes. Then, the bound water in it removes, which reduces the bonding of alkali-activated blast furnace slag powder and reducing the mechanical strengths. Compared with Aydin’s research, the flexural strength and compressive strength of alkali-activated blast furnace slag powder measured at the temperature of 800 °C with assembly unit of CaO and MgO are increased by 24.1% and 13.2% [24].

**Figure 11:**
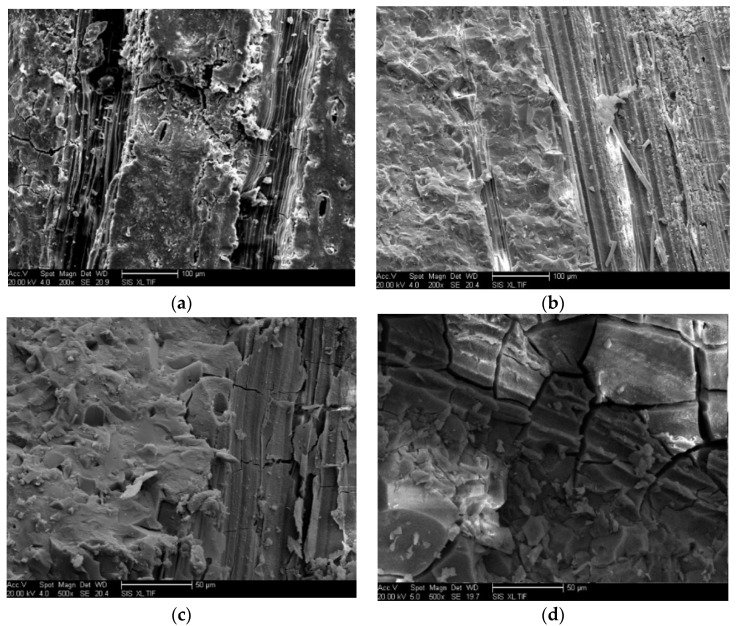
The SEM morphology of alkali-activated blast furnace slag powder measured at high temperature environment. (**a**) 200 °C. (**b**) 400 °C. (**c**) 600 °C. (**d**) 800 °C.

Figure 11 shows the scanning electron microscope photos of alkali-activated blast furnace slag powder measured at high temperatures of 200 °C, 400 °C, 600 °C and 800 °C. As illustrated in Figure 11, the fibrous hydration products can be found in the scanning electron microscope photos. When the temperature is higher than 400 °C, more cracks are observed. The SEM photos further exhibit that the mechanical strength of alkali-activated blast furnace slag powder is decreased by higher measuring temperature.

## 4. Conclusions

The mechanical strength and drying shrinkage rate of alkali-activated blast furnace slag powder are researched. The conclusions are acquired as follows.

The water-binder ratio of the alkali-activated blast furnace slag powder demonstrated negative effect on the following flexural and compressive strengths. Specifically, the mechanical strengths increase with an increasing curing age and a lower water-binder ratio. Meanwhile, the mechanical strengths decrease with the increasing temperature.

The addition of MgO and CaO leads to improve the mechanical strengths of alkali-activated blast furnace slag powder. While the hydrolysis rate of CaO is faster than that of MgO.

A higher water-binder ratio leads to increasing the drying shrinkage rate. The activated blast furnace slag powder with CaO shows a higher drying shrinkage rate. The measuring temperature can lead to decreasing the mechanical strengths of activated blast furnace slag powder.

## Figures and Tables

**Table 1:** Main chemical composition of raw materials (%).

Types	SiO_2_	AL_2_O_3_	CaO	MgO	Fe_2_O_3_	Na_2_O	Loss on Ignition
Blast furnace slag powder	36.9	15.66	37.57	9.3	0.36	0.57	-
Active MgO	7.32	-	2.02	88.36	-	0.01	1.88
Expansive agent	15.92	4.12	70.80	-	-	-	5.69

**Table 2:** The mixing proportions of alkali-activated blast furnace slag powder.

Types	BFS (kg)	MgO (kg)	CaO (kg)	Water-Binder Ratio	Fiber Volume (%)	Fiber Treatment Method
W/B-0.42	1584	/	/	0.42	/	/
W/B-0.42-F-1%	1584	/	/	0.42	1	/
W/B-0.42-WF-1%	1584	/	/	0.42	1	Water treatment
W/B-0.42-M-10%	1425.6	158.4	/	0.42	/	/
W/B-0.42-M-20%	1267.2	316.8	/	0.42	/	/
W/B-0.42-M-30%	1108.8	475.2	/	0.42	/	/
W/B-0.42-M-20%-C-10%	1108.8	316.8	158.4	0.42	/	/
W/B-0.42-M-10%-C-20%	1108.8	158.4	316.8	0.42	/	/
W/B-0.42-M-0%-C-30%	1108.8	/	475.2	0.42	/	/
W/B-0.35	1584	/	/	0.35	/	/
W/B-0.35-F-1%	1584	/	/	0.35	1	/
W/B-0.35-WF-1%	1584	/	/	0.35	1	Water treatment
W/B-0.35-M-10%	1425.6	158.4	/	0.35	/	/
W/B-0.35-M-20%	1267.2	316.8	/	0.35	/	/
W/B-0.35-M-30%	1108.8	475.2	/	0.35	/	/
W/B-0.35-M-20%-C-10%	1108.8	316.8	158.4	0.35	/	/
W/B-0.35-M-10%-C-20%	1108.8	158.4	316.8	0.35	/	/
W/B-0.35-M-0%-C-30%	1108.8	/	475.2	0.35	/	/
W/B-0.42-M-30%-WF-1%	1108.8	475.2	/	0.42	1	Water treatment
W/B-0.42-M-20%-C-10%-WF-1%	1108.8	316.8	158.4	0.42	1	Water treatment
W/B-0.35-M-30%-WF-1%	1108.8	475.2	/	0.35	1	Water treatment
W/B-0.35-M-20%-C-10%-WF-1%	1108.8	316.8	158.4	0.35	1	Water treatment

## Data Availability

The data used to support the findings of this study are available from the corresponding author upon request.
